# An H-NS Family Protein, Sfh, Regulates Acid Resistance by Inhibition of Glutamate Decarboxylase Expression in *Shigella flexneri 2457T*

**DOI:** 10.3389/fmicb.2017.01923

**Published:** 2017-10-05

**Authors:** Chang Niu, Dongshu Wang, Xiaoqing Liu, Hongsheng Liu, Xiankai Liu, Erling Feng, Chao Pan, Ruifeng Wang, Wei Xiao, Xingming Liu, Xinrui Liu, Li Zhu, Hengliang Wang

**Affiliations:** ^1^Department of Biochemistry, College of Life Sciences, Capital Normal University, Beijing, China; ^2^State Key Laboratory of Pathogen and Biosecurity, Beijing Institute of Biotechnology, Beijing, China

**Keywords:** *S. flexneri*, blue native-PAGE, 2-DE, glutamate-dependent acid-resistance systems, H-NS family member

## Abstract

The glutamate-dependent acid-resistance system is the most effective acid tolerance pathway in *Shigella*, allowing survival in extremely acidic environments. However, the regulation of this system in *Shigella* remains elusive. In the current study, we identified significant differences in the levels of glutamate decarboxylase between three *Shigella flexneri* strains with different levels of acid resistance using blue native-polyacrylamide gel electrophoresis (PAGE) and isoelectric focusing (IEF)/sodium dodecyl sulfate polyacrylamide gel electrophoresis (SDS-PAGE) analysis. The results showed that the degree of acid resistance and the levels of GadA/B were significantly lower in strain 2457T compared with two other *S. flexneri* strains. It has been reported that plasmid pSf-R27 is expressed in strain 2457T but not in the other 142 sequenced *S. flexneri* isolates. pSf-R27 encodes protein Sfh, which belongs to a family of histone-like nucleoid-structuring (H-NS) proteins that participate in the transcriptional control of glutamate-dependent acid resistance, implicating pSf-R27 in the lower acid resistance of strain 2457T. Transformation of pSf-R27 or *sfh* alone into strain 301 resulted in decreased expression of GadA/B in the recombinant strains. Thus, we confirmed that H-NS family protein Sfh, bound to the *gadA/B* regulatory region and regulates the expression of glutamate decarboxylase at the transcriptional level. We also examined the acid tolerance of the wild-type and recombinant strains using flow cytometry and determined that the acid tolerance of *S. flexneri* is closely related to the expression of GadA/B. These findings further our understanding of the acid tolerance of *S. flexneri*, especially via the glutamate-dependent pathway.

## Introduction

Shigellosis, a dysentery disease caused by *Shigella* bacteria, is an important cause of morbidity and mortality, especially in children under 5 years of age in developing countries (Bennish et al., 1990; Rahman et al., 2011). Further, a recent study of epidemiological and microbiological data in Asia found that *Shigella* DNA was detected in one-third of *Shigella*-negative diarrheal samples, indicating that the incidence of this disease may even exceed current estimates (von Seidlein et al., 2006). With consistently high incidence rates and the emergence of multidrug-resistant *Shigella* strains, shigellosis is a critically important global health problem (Sansonetti, 2006). In China, a retrospective review indicated that *Shigella flexneri* was responsible for 86% of shigellosis episodes between 1991 and 2000, and that the predominant serotype was *S. flexneri* 2a (80%) (Wang et al., 2006).

*Shigella* species are highly virulent, with as few as 10 bacterial cells capable of causing disease. Shigellosis has an incubation period of 12–48 h, and may last for up to a week (DuPont et al., 1989; Ashkenazi and Cohen, 2013). A high level of acid tolerance is essential for bacteria to survive in the low pH environment of the stomach, which is necessary for invasion of the intestinal mucosa (Sansonetti et al., 1982). In general, intestinal bacteria must possess one or more of five acid-resistance (AR) pathways, AR1–5, to survive the acidic environment of the stomach. AR1–5 are mediated by cyclic adenosine monophosphate (cAMP) receptor protein, glutamate decarboxylases, arginine decarboxylases, lysine decarboxylases, and ornithine decarboxylase, respectively (Kashiwagi et al., 1992; Foster, 2004). In *Shigella*, AR1 and AR2 are the main acid-tolerance pathways (Bhagwat and Bhagwat, 2004) allowing survival in extremely acidic environments (Sherburne et al., 2000), with AR2 (glutamate-dependent AR, GD-AR) being particularly important. The glutamate decarboxylase system encompasses three genes: *gadA, gadB*, and *gadC*. *GadA*, and *gadB* encode highly homologous glutamate decarboxylase isoforms (Smith et al., 1992), while *gadC* encodes a putative inner-membrane glutamate-γ-aminobutyric acid (GABA) antiporter. GadA and GadB are pyridoxal 5′-phosphate-dependent enzymes that convert the α-decarboxylation product of L-glutamate to GABA and carbon dioxide, consuming a cytoplasmic proton in the process (Bearson et al., 1997). GABA is transported out of the cell via the inner membrane antiporter GadC in exchange for new substrate, thus preventing the internal pH from depressing to lethal levels. In this way, protons leaking into the cell during acid stress are consumed and excreted from the cell. Previous research has shown that *gadB* and *gadC* form an operon (De Biase and Pennacchietti, 2012), the expression of which is influenced by a variety of factors, including growth phase, osmotic pressure, and oxygen. For example, the expression of *gadB* is inhibited by histone-like nucleoid-structuring (H-NS) protein during log phase growth, which is removed by RpoS in stationary phase (Castanie-Cornet et al., 1999). However, a more detailed picture of the mechanisms and regulatory network of the GD-AR system is not yet available.

To date, whole genome sequencing of multiple *S. flexneri* strains has been completed (Venkatesan et al., 2001; Jin et al., 2002). Interestingly, the *Salmonella enterica* serovar Typhi R27-like plasmid pSf-R27 has only been identified in one strain (2457T) out of the 142 sequenced *S. flexneri* isolates, including 57 serotype 2a strains (Wei et al., 2003). pSf-R27 was implicated in the accumulation and spread of antibiotic resistance (Sherburne et al., 2000); however, the exact function of this plasmid in strain 2457T remains unknown.

In the current study, we compared the acid resistance of three wild-type *S. flexneri* stains and carried out a proteomic analysis to investigate differences in protein expression. The expression patterns of protein complexes and monomers were analyzed using optimized blue native-polyacrylamide gel electrophoresis (BN-PAGE) and isoelectric focusing (IEF)/sodium dodecyl sulfate polyacrylamide gel electrophoresis (SDS-PAGE) two-dimensional gel electrophoresis systems. Matrix-assisted laser desorption/ionization time-of-flight mass spectrometry (MALDI-TOF MS) identified significant differences in the expression of GadA/B, with levels of these proteins significantly lower in strain 2457T compared with the other two strains. Transformation of pSf-R27 into strain 301 resulted in decreased expression of GadA/B in the recombinant strain. Further experiments showed that H-NS family protein Sfh, which is present on plasmid pSf-R27, bound to the *gadA/B* regulatory region, and that expression of *sfh* in strain 301 also inhibited expression of GadA/B. We also examined the acid tolerance of the wild-type and recombinant strains using flow cytometry and determined that the acid tolerance of *S. flexneri* is closely related to the expression of GadA/B. These findings further our understanding of the acid tolerance of *S. flexneri*, especially via the glutamate-dependent pathway.

## Materials and methods

### Bacterial strains and growth conditions

*Escherichia coli* strain DH5α was used for plasmid construction and was maintained on Luria-Bertani (LB) agar or broth (Difco) at 37°C. Wild-type *S. flexneri* serotype 2a strains 301 and 2457T, and serotype 5a strain M90T, were grown on tryptic soy agar (Difco) containing 0.01% (w/v) Congo red or in LB broth at 30 and 37°C. When necessary, nalidixic acid (50 μg/mL), streptomycin (50 μg/mL), or chloramphenicol (30 μg/mL) were added to the growth media.

### Construction of transconjugant and mutant *S. flexneri* strains

As shown in Supplementary Figure S1, gene fragment *R0139*, amplified from *S. flexneri* strain 2457T using primers R0139-1 and R0139-2 (Supplementary Table S1), was ligated into chloramphenicol resistance-conferring *pir*-dependent suicide vector pXL275, generating recombinant plasmid pXL275-R0139. pXL275-R0139 was then transformed into *E. coli* S17-λ*pir*, and transferred into *S. flexneri* strain 2457T by conjugation according to the method of Klümper et al. (2014). Following homologous recombination into plasmid pSf-R27 at the *R0139* site, the resulting pSf-R27 plasmid contained the chloramphenicol resistance marker. Using helper plasmid pRK2013, the recombinant pSf-R27 plasmid was transferred into *S. flexneri* strain 301, as described previously (Zhang et al., 2013). Resulting transconjugants were named 301/pSf-R27, and were purified and used for further analyses.

Recombinant plasmid pAK-sfh was prepared by ligating *sfh*, amplified from *S. flexneri* strain 2457T using primers sfh-1 and sfh-2 (Supplementary Table S1), into low-copy-number vector pAK (Zhao et al., 2010). The recombinant plasmid was then transformed into electrocompetent *S. flexneri* 301 cells using a Bio-Rad electroporation apparatus to generate strain 301/pAK-sfh.

### Acid tolerance assay

Bacteria were grown to stationary phase at 37°C in LB medium adjusted to pH 5.0. Aliquots (1 mL) of culture were then centrifuged for 5 min at 2,000 × *g*, and the resulting pellets resuspended in 1-mL volumes of LB medium adjusted to pH 2.5 or pH 5.0 (Lin et al., 1995; Yang et al., 2015). The suspensions were incubated for 30 min at 37°C with shaking at 220 rpm. Bacterial cells were then collected by centrifugation as described above, and the resulting pellets washed three times with PBS. Cells were then stained using a Cell Viability Kit (BD Biosciences) for 15 min and measured using a FACScan flow cytometer (BD Biosciences). The data were analyzed using CellQuest software. Two or three repetitions were performed for each experiment.

### Two-dimensional page (2-DE) and data analysis

Preparation of whole-cell protein extracts (complex and monomer samples) and 2-DE analysis (BN-PAGE and IEF/SDS-PAGE) was performed as previously described (Zhu et al., 2010a; Niu et al., 2013).

### Protein identification by MALDI-TOF/TOF

All of the protein spots generated by 2-DE were analyzed by MALDI-TOF/TOF MS. The protein spots were carefully excised from the gel, destained using destaining solution (50% acetonitrile, 25 mM acid ammonium carbonate), and then digested for 13 h using sequencing-grade modified trypsin (Roche). Peptides from the digested proteins were used for MALDI-TOF/TOF analysis. MALDI-TOF MS was performed using an Ultraflex^III^ MALDI-TOF mass spectrometer (Bruker Daltonics) operating in reflectron mode with 20 kV accelerating voltage and 23 kV reflecting voltage. A saturated solution of α-cyano-4-hydroxycinnamic acid in 50% acetonitrile and 0.1% trifluoroacetic acid was used as the matrix. A 1-μL volume of a mixture of the matrix and sample solutions at a 1:1 ratio was applied to the Score384 target well. The SNAP algorithm (S/N threshold: 5; Quality Factor Threshold: 30) in FlexAnalysis v.2.4 was used to select the 150 most prominent peaks in the mass range m/z 700–4,000. The subsequent MS/MS analysis was performed in a data-dependent manner, and the 10 most abundant ions were subjected to high energy collision-induced dissociation analysis. The collision energy was set to 1 keV, and nitrogen was used as the collision gas.

### Data interpretation and database searching

To deal with one PMF and multiple TOF/TOF spectra from one sample as a single combined dataset, the raw data were first merged into one MGF file using Biotools v3.0 software, and then searched using Mascot 2.1 (Matrix Science Ltd.) against the *S. flexneri* 2a 2457T genome database to eliminate redundancy resulting from multiple members of the same protein family. The database contains 4,540 entries, including all of the predicted open reading frames on the chromosome of *S. flexneri* 2a 2457T (GenBank GI:30043918), the virulence plasmid pCP301 (GenBank GI:18462515) from *S. flexneri* 2a 301, and the large IncHI plasmid R27 (GenBank GI:7800243) from *Salmonella* Typhi. Results were checked against the NCBInr database (version 20061021, 4,072,503 sequences) to eliminate known contaminants. The search parameters used were as follows: trypsin digestion with one missed cleavage; carbamidomethyl modification of cysteine as a fixed modification and oxidation of methionine as a variable modification; peptide tolerance maximum, ±100 ppm; MS/MS tolerance maximum, ±0.6 Da; peptide charge, +1; monoisotopic mass. Scores >21 are considered significant (*P* < 0.05) for a local MS/MS search. For unambiguous identification of proteins, more than five peptides must be matched.

### RNA isolation and preparation of cDNA

For quantitative reverse-transcriptase polymerase chain reaction (qRT-PCR) analysis, bacteria were cultured at 37°C and harvested in stationary phase. Total RNA was isolated from the cultures using RNeasy Mini Spin Columns (Qiagen) and treated with RNase-free DNase I (New England Biolabs) according to the manufacturer's instructions. cDNA was generated from 3 μg of each RNA sample using a Revert Aid First Strand cDNA Synthesis Kit (Thermo Scientific).

### qRT-PCR analysis

qRT-PCR analysis was carried out using an iCycler iQ Real-Time PCR System (BioRad) in 50-μL reaction mixtures containing 1 μL of cDNA, 600 nM of each primer, and iQ SYBR Green Supermix (BioRad) according to the manufacturer's instructions. Reactions were carried out under the following conditions: 30 s at 95°C, followed by 40 cycles of 15 s at 95°C, 30 s at the specific annealing temperature, and 30 s at 72°C. The qRT-PCR reactions were performed in triplicate for each of the three biological replicates tested. Data were analyzed using CFX Manager 2.1 (BioRad). Gene-specific primers (Supplementary Table S1) were designed using Primer Premier 5.0 software (Premier Biosoft). Relative amounts of cDNA were normalized to the amounts of 16S rRNA cDNA in each sample. Results represent the mean from at least three independent experiments.

### Purification and identification of sequence-specific DNA binding proteins

End-biotinylated primer sets gadAp1/gadAp2 and gadBp1/gadBp2 were designed to amplify the *gadA* and *gadB* DNA regulatory regions, respectively (Supplementary Table S1). Using DNA extracted from wild-type strain 301 as a template, the biotin-labeled *gadB* DNA regulatory region was amplified and then coupled to Dynabeads M-280 Streptavidin (Invitrogen) according to the manufacturer's instructions. An end-biotinylated DNA fragment, which does not bind potential target proteins, was also included as a negative control. Approximately 15 mL of an overnight bacterial culture were then harvested, resuspended in 1 mL of TGED buffer (20 mM Tris-HCl, pH 8.0, 1 mM EDTA, 10% (v/v) glycerol, 1 mM DTT, 0.01% Triton X-100) containing 100 mM NaCl, and then lysed by sonication. The DNA-coupled magnetic beads were incubated with the cell lysate for 30 min at room temperature in TGED buffer containing 100 mM NaCl, and then washed three times in the same buffer. Proteins were eluted on ice in 50 μL of TGED buffer containing 1 M NaCl. Following purification using a 2-D Clean-Up Kit (GE Healthcare), proteins were pipetted into 10 kDa ultrafiltration tubes (Amicon Ultra-0.5; Millipore) and centrifuged at 13,000 × *g* for 5 min. The flow-through was discarded and 0.1% (m/v) DTT in 50 mmol/L NH_4_HCO_3_ was added to the ultrafiltration tubes. Samples were incubated for 30 min and then centrifuged as described above. The flow-through was again discarded and 0.25% (m/v) IAA in 50 mmol/L NH_4_HCO_3_ was added to the tubes and incubated for 30 min in the dark. The samples were centrifuged as described above and then washed at least three times with 50 mmol/L NH_4_HCO_3_ (the flow-through was discarded after each centrifugation step).

The resulting proteins were digested with trypsin (Roche, 1:50, w/w) for 16 h at 37°C, and flow-through peptides were collected in a new tube for liquid chromatography tandem mass spectrometry (LC-MS/MS) analysis. Peptides were separated on a 25-cm C18 nano column using EASY-nLC (Thermo Fisher Scientific). Samples were loaded and then eluted for 30 min using a 4–90% ACN fraction-optimized non-linear gradient in 0.1% formic acid. Eluted peptides were detected using an Orbitrap Fusion Lumos Tribrid mass spectrometer (Thermo Fisher Scientific), and then semi-quantified using a spectral counting approach as described previously (Gao et al., 2015). Each sample was independently analyzed three times, and all spectra were compared against the *S. flexneri* 2a 2457T database described above using PD v.2.1 software (Thermo Fisher Scientific).

### Electrophoretic mobility shift assay (EMSA)

His-tagged HNS and Sfh proteins were expressed in *E. coli* BL21 (DE3) cells using the pET28a vector. The corresponding gene regions were amplified from 2457T using primers hnsp1/hnsp2 and sfhp1/sfhp2, respectively. *gadA* (350 bp) and *gadB* (450 bp) DNA regulatory region fragments were amplified by PCR using 5′-end-biotinylated primers. All primer sequences are listed in Supplementary Table S1. The PCR fragment was gel-purified twice using a High Pure PCR Product Purification Kit (Roche). The EMSA assay was conducted using a LightShift Chemiluminescent EMSA Kit and a Chemiluminescent Nucleic Acid Detection Module (Thermo Fisher Scientific) as per the manufacturer's instructions.

### Western blot analysis

The SDS polyacrylamide gels were transferred to PVDF membranes at 15 V for 1.5 h. The resulting PVDF membranes were blocked with 10% (w/v) skim milk powder in TBS (100 mmol/l Tris-HCl, pH 7.5, 0.9% (w/v) NaCl) containing 0.1% (v/v) Tween 20 (TBST) for 1 h. Membranes were incubated with anti-GadB antibody (Abmart Corp.) diluted in TBST for 1–2 h at room temperature or at 4°C overnight at the recommended concentration, followed by detection using ECL reagents (Thermo Fisher Scientific) and manual film development.

## Results

### Differential expression of GadA/B in 2457T and other *S. flexneri* strains

**Figure 1:**
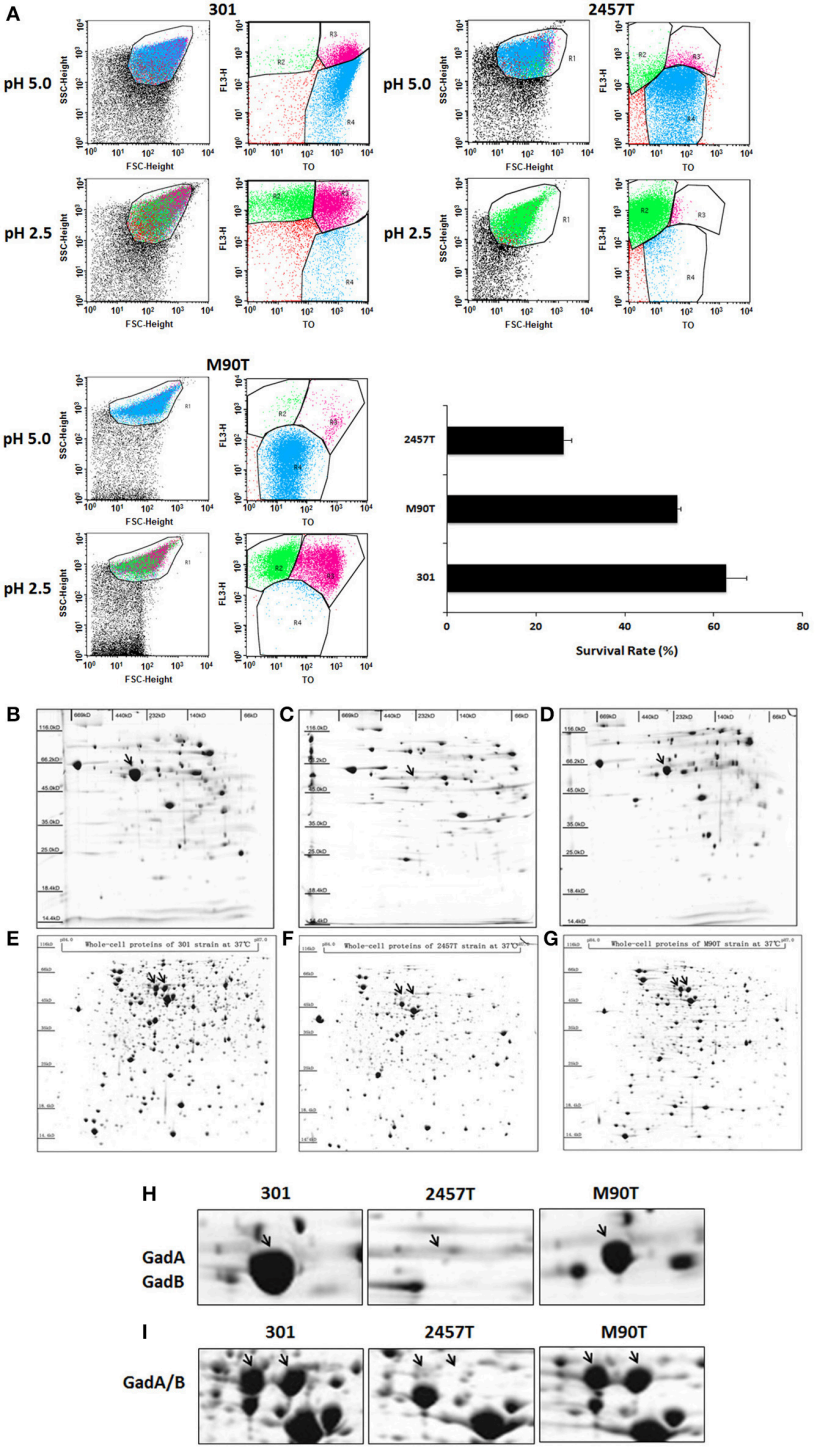
Analysis of acid resistance and differential expression of GadA/B in *Shigella flexneri* strains 301, 2457T, and M90T. **(A)** The survival rates of bacterial cells following culture in acid medium were detected by flow cytometry. The column diagram results are shown as means ± *SD* of two independent experiments. R1 represented the whole cells; R2–R4 were set around the dead, injured, and live bacterial populations, respectively. **(B–G)** Whole-cell soluble protein complexes or protein subunits from *S. flexneri* strains 301 **(B,E)**, 2457T **(C,F)**, and M90T **(D,G)** separated by blue-native polyacrylamide gel electrophoresis or isoelectric focusing/sodium dodecyl sulfate polyacrylamide gel electrophoresis. **(H)** Enlarged images of the GadA/B protein complex and **(I)** GadA and GadB protein subunit spots.

To examine the molecular mechanism of acid resistance in *S. flexneri*, we compared the acid resistance of three different strains. Strains 2457T and 301 were identified as serotype 2a and showed ~98% genome homology, while M90T is a serotype 5a strain. The stress tolerance of the strains was examined using survival assays, which showed that the survival rate of 2457T was obviously lower than those of the other two *S. flexneri* strains at pH 2.5 (Figure 1A).

Because the glutamic acid decarboxylase system is the most effective acid-tolerance pathway in *Shigella*, we examined the expression levels of soluble protein complexes among the three strains. Strains were grown aerobically in 100 mL of LB medium at 37°C, and then the protein complexes were separated and analyzed by BN-PAGE 2-DE. Compared with strains 301 and M90T, the abundance of the GadA/B complex in strain 2457T was significantly reduced (Figures 1B–D,H). IEF/SDS-PAGE was then performed to analyze the expression levels of protein monomers. Whole cell protein extracts were separated using IPG strips with a linear gradient of pH 4–7 in the first dimension, and results showed a corresponding decrease in the levels of GadA and GadB in strain 2457T (Figures 1E–G,I).

### Plasmid pSf-R27 inhibits the expression of GadA/B in strain 301

**Figure 2:**
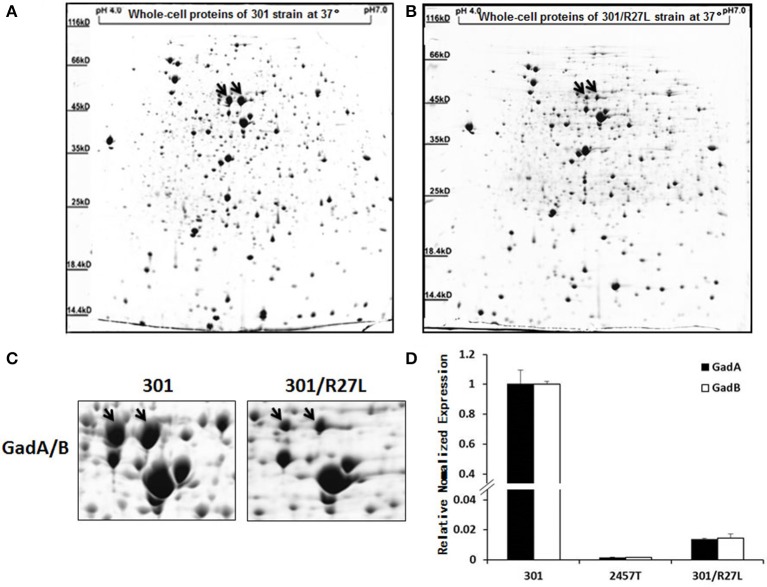
Analysis of soluble proteins of strains 301 **(A)** and 301/pSf-R27 **(B)** by isoelectric focusing/sodium dodecyl sulfate polyacrylamide gel electrophoresis. **(C)** Enlarged images of GadA/B protein subunit spots. **(D)** Transcript levels of *gadA/B* were determined by quantitative reverse-transcriptase polymerase chain reaction analysis normalized to the levels of the 16S rRNA gene in each sample. The column diagram results are shown as the means ± *SD* of three independent experiments.

Plasmid pSf-R27 is present in strain 2457T but not in strain 301 (Supplementary Figure S2) (Wei et al., 2003). To determine whether pSf-R27 is responsible for the differences in GadA/B expression between the two strains, pSf-R27 was transformed into strain 301. As expected, IEF/SDS-PAGE showed that levels of GadA and GadB expression were significantly decreased in recombinant strain 301/pSf-R27 (Figures 2A–C), suggesting that pSf-R27 inhibits the expression of GadA and GadB. To verify the proteomic data, the effects of pSf-R27 on *gadA* and *gadB* mRNA levels were quantitated using qRT-PCR analysis. Consistent with the protein expression data, pSf-R27 reduced the levels of *gadA* and *gadB* mRNA in strain 301 (Figure 2D).

### H-NS family proteins bind to the regulatory region of *gadA/B*

To clarify the mechanism of pSf-R27 regulation of GadA/B expression, the region upstream of *gadA*/*B* was amplified using biotinylated primers and then linked to streptavidin-coated magnetic beads to capture interacting proteins in *S. flexneri* strains 2457T and 301. Captured proteins were identified by LC-MS/MS. Information regarding the 10 most abundant proteins is provided in Table 1. According to the MS data, three H-NS family members (Sfh, H-NS, and StpA) were identified. Protein Sfh, the coding sequence for which is located on plasmid pSf-R27, was present at a higher abundance (semi-quantified by PSM numbers) in strain 2457T than the well-known regulator StpA, which belongs to the H-NS protein family (Zhang and Belfort, 1992). StpA also has a similar structure and function to H-NS (Sonnenfield et al., 2001; Deighan et al., 2003), which is a selective binding protein that represses *gadA*/*B* transcription and expression in *E. coli* (Heath and Rock, 1996). Identification of H-NS binding to the *gadA*/*B* promoter in *S. flexneri* confirmed the applicability of our experiments.

**Table 1:** Identification of DNA-binding proteins by LC-MS/MS.

**Accession**	**Gene**	**Synonym**	**Sum PEP score**	**MW (kDa)**	**calc. pI**	**PSMs in samples**																	
						**301_control**			**301_gadA**			**301_gadB**			**2457T_control**			**2457T_gadA**			**2457T_gadB**		
10957353	*Sfh*	R27_p164	98.50470579	15.199	5.48	0	1	0	0	2	0	0	3	2	0	2	3	19	362	158	23	553	210
30041005	*hns*	S1323	75.04501874	15.53	5.47	0	7	0	4	213	35	2	188	23	0	12	5	21	428	54	26	525	58
30042989	*rpoC*	S3674	56.47587314	155.063	7.08	0	43	0	0	60	0	0	39	0	0	1	8	6	44	84	6	47	96
30042990	*rpoB*	S3675	51.62526747	150.538	5.26	1	12	0	0	26	0	1	27	0	1	0	4	9	26	49	9	37	46
30039923	*aceF*	S0114	47.81738081	65.805	5.17	4	17	34	4	48	20	19	23	18	2	4	6	2	24	24	3	21	37
30042293	*stpA*	S2883	44.35543658	15.338	8.4	0	1	0	1	17	1	0	12	2	0	1	2	13	145	91	10	146	123
30040383	*seqA*	S0617	42.58132432	20.303	8.7	0	0	0	0	1	0	0	0	0	0	0	0	2	0	16	1	0	16
30042130	*ppk*	S2694	35.94933989	80.381	8.92	0	6	0	3	5	10	3	8	9	3	4	9	2	5	29	7	5	13
30041044	*topA*	S1361	33.54348311	97.305	8.35	0	4	2	1	9	4	1	7	11	0	8	0	8	12	32	10	16	42
30040186	*hupB*	S0391	29.27470057	9.22	9.7	0	0	0	0	2	3	0	4	3	1	0	0	2	4	4	2	4	12

*PSM, peptide spectrum match; PEP, posterior error probability; 301_contro, the sample using end-biotinylated negative control DNA fragment to pull down DNA binding proteins in 301 strain protein extracts; 301_gadA, the sample using end-biotinylated gadA regulatory region DNA fragment to pull down DNA binding proteins in 301 strain protein extracts; 301_gadB, the sample using end-biotinylated gadB regulatory region DNA fragment to pull down DNA binding proteins in 301 strain protein extracts; 2457T_contro, the sample using end-biotinylated negative control DNA fragment to pull down DNA binding proteins in 2457T strain protein extracts; 2457T_gadA, the sample using end-biotinylated gadA regulatory region DNA fragment to pull down DNA binding proteins in 2457T strain protein extracts; 2457T_gadB, the sample using end-biotinylated gadB regulatory region DNA fragment to pull down DNA binding proteins in 2457T strain protein extracts*.

**Figure 3:**
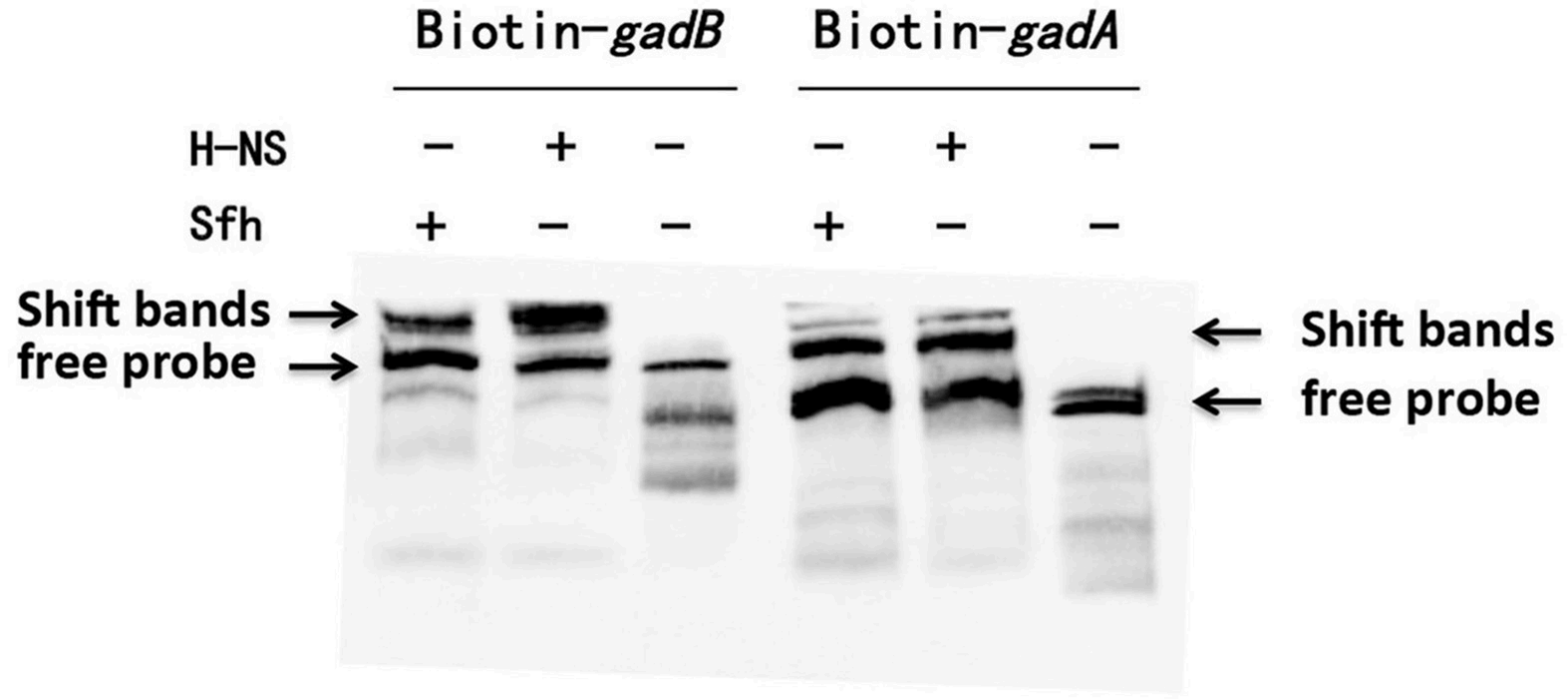
Verification of the interaction of Sfh with *gadA*/*B* regulon-specific DNA. Electrophoretic mobility shift assays were carried out with *gadA*/*B* regulatory region DNA fragments. The upper arrows indicate the position of the free probe and Sfh-DNA/HNS-DNA complexes, and the lower arrows indicate the position of the free probe.

We further examined the effects of Sfh binding to the *gadA/B* regulatory region *in vitro* by electrophoretic mobility shift assays. The results indicated that the *gadA/B* sequence was shifted by H-NS and Sfh (Figure 3), which is in keeping with the proposed binding of Sfh upstream of the *gadA/B* sequence.

### H-NS family member Sfh inhibits the expression of GadA/B in strain 301

**Figure 4:**
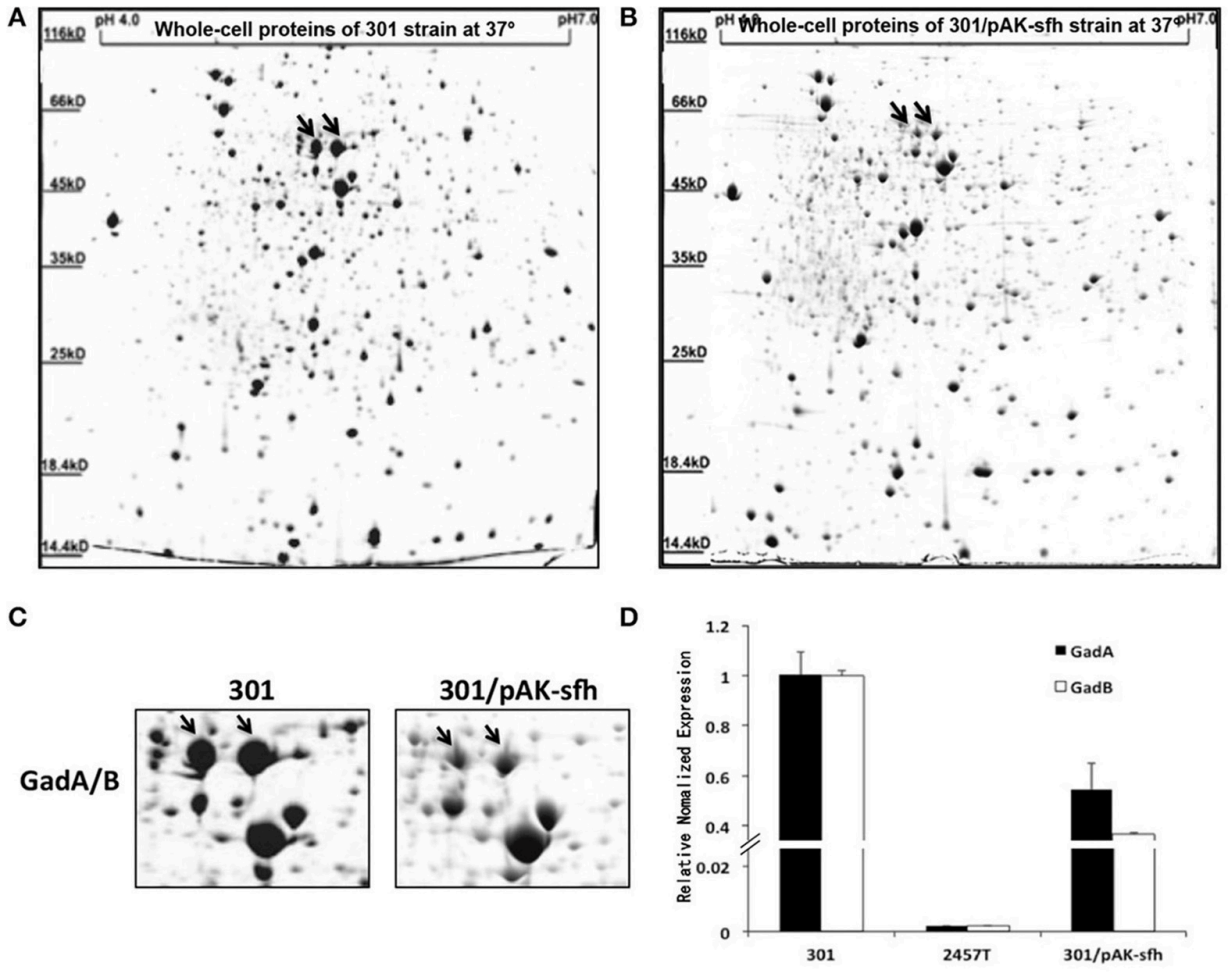
Analysis of soluble proteins from strains 301 **(A)** and 301/pAK-sfh **(B)** by isoelectric focusing/sodium dodecyl sulfate polyacrylamide gel electrophoresis. **(C)** Enlarged images of GadA/B protein subunits spots. **(D)** Transcript levels of *gadA*/*B* were determined by quantitative reverse-transcriptase polymerase chain reaction analysis normalized to the levels of the 16S rRNA gene in each sample. The column diagram results are shown as means ± *SD* of three independent experiments.

Because Sfh is located on plasmid pSf-R27 and was shown to bind to the regulatory region of *gadA/B*, we examined whether Sfh regulates the expression of GadA/B using IEF/SDS-PAGE analysis. As expected, the expression of GadA/B was reduced in strain 301/pAK-sfh (strain 301 expressing recombinant plasmid pAK-sfh) (Figures 4A–C). Moreover, qRT-PCR analysis indicated that Sfh inhibits the expression of *gadA*/*B* at the transcriptional level (Figure 4D).

### Sfh regulates the acid tolerance of strain 301

**Figure 5:**
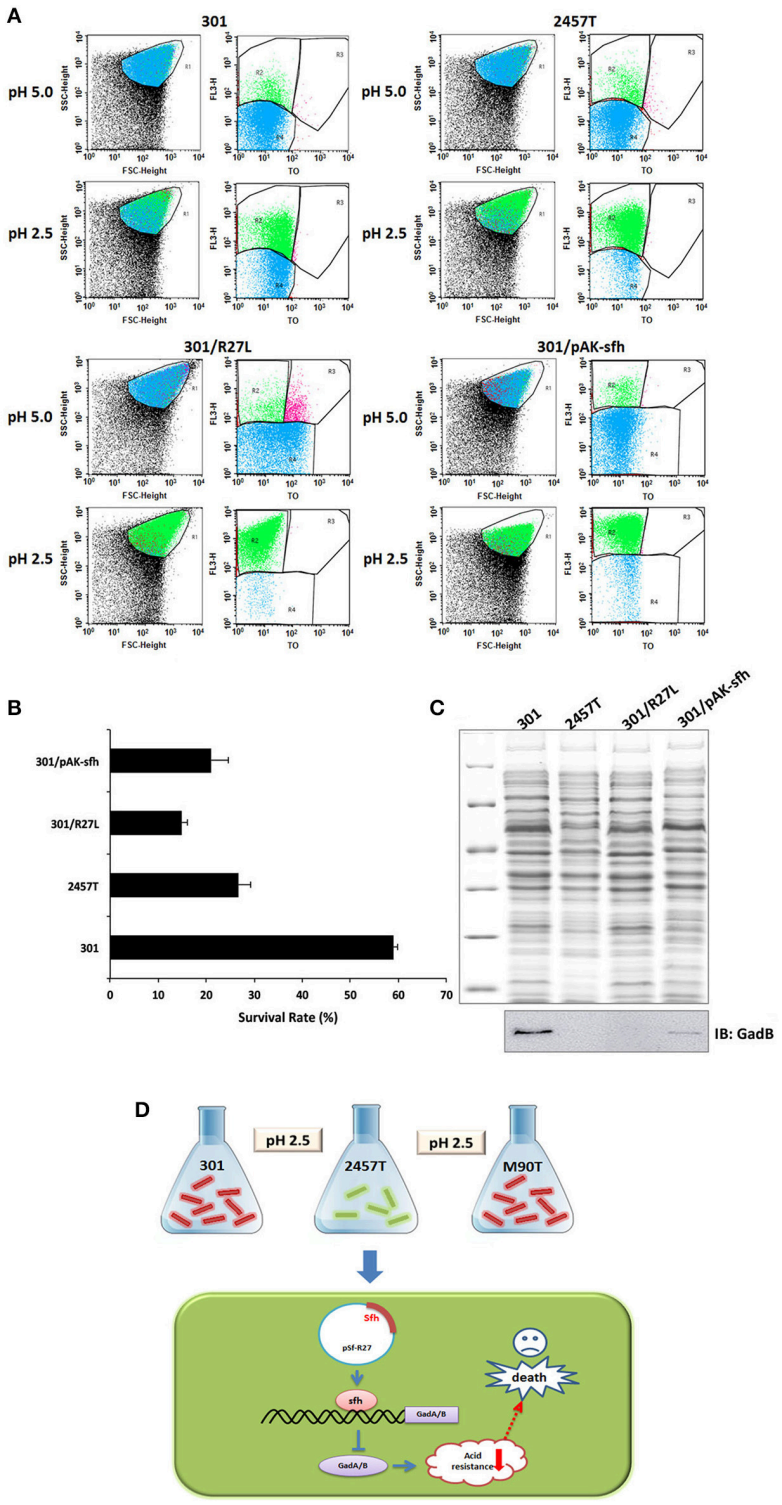
Flow cytometry analysis of *Shigella flexneri* acid tolerance. **(A)** Flow cytometry was used to provide counts of living cells before and after acid treatment, calculate the viability of bacterial cells, and then infer the strength of the acid tolerance. R1 represented the whole cells; R2–R4 were set around the dead, injured, and live bacterial populations, respectively. **(B)** Survival rates of bacterial cells following culture in acid medium. The column diagram results are shown as means ± *SD* of two independent experiments. **(C)** Detection of GadB in protein samples using western blot. The upper figure shows total protein gel staining, and the lower figure shows bands immunoblotted with GadB antibody. **(D)** A schematic model of regulation of Sfh on acid resistance in *S. flexneri*.

Because GadA and GadB play important roles in acid resistance, we examined whether plasmid pSf-R27, and Sfh in particular, regulates acid tolerance in *S. flexneri*. Survival of strains 301 and 2457T was examined following growth at pH 2.5 and compared with that at pH 5.0 using flow cytometry. As expected, the acid tolerance of strain 301 was significantly higher than that of strain 2457T. Further, the presence of plasmid pSf-R27 and the expression of Sfh reduced the acid tolerance of strain 301 (Figures 5A,B).

We also examined the expression of GadB in each strain using western blot analysis, and the results were mainly consistent with those generated by IEF/SDS-PAGE analysis (Figure 5C). The expression of GadB in strain 2457T was significantly lower than that in strain 301, and the presence of plasmid pSf-R27 or expression of Sfh in strain 301 reduced the detectable levels of GadB. These results indicated that the acid tolerance of *S. flexneri* is closely related to the expression levels of GadA/B.

## Discussion

Many factors are involved in the transcriptional control of GD-AR under different growth conditions, including RpoS (the stationary phase-specific alternative RNA polymerase subunit σ^s^) (Bhagwat and Bhagwat, 2004), the global transcription regulator H-NS (Giangrossi et al., 2005), and cAMP systems (Foster, 2004). RpoS induces the transcription of *gadX*, which activates *gadAB* expression and simultaneously turns off the expression of other virulence-related genes (Tramonti et al., 2002). Binding of cAMP to the receptor protein CRP affects the GD-AR system by repressing *rpoS* transcription (Ma et al., 2002, 2003), while H-NS also directly suppresses *gadA* and *gadBC* expression (Tramonti et al., 2002; Waterman and Small, 2003). To confirm which of these systems affects the expression of GadA/B in strain 2457T, we generated strain 2457T/RpoS^301^ (2457T transformed with *rpoS* from 301) and found that GadA/B expression was unchanged (data not shown). In general, H-NS-dependent regulation mainly occurs at a transcriptional level. Transcriptional repression is the result of preferential binding by H-NS to promoter regions, leading to inactivation of the RNA polymerase. It has been reported that the *E. coli* H-NS protein can prevent sigma factor 70 from carrying out its transcriptional function and can inactivate RpoS, thereby inhibiting *gadA*/*B* transcription (Zhang and Belfort, 1992). Therefore, further research is needed into the role of Sfh in *gadA*/*B* transcriptional repression.

Acid resistance is an important feature for intestinal pathogenic bacteria as they must survive in the presence of gastric acid and unstable fatty acids produced by the gut (Sansonetti et al., 1982). The acid stress response systems of intestinal bacteria rely on enzymes and molecular chaperones, with the three main acid resistance mechanisms based on glutamic acid, arginine, and the lysine decarboxylase pathway. These systems form a complex regulatory network, allowing bacteria to survive in extremely acidic environments. However, *S. flexneri* has a lysine decarboxylase deficiency, and arginine transport is inhibited (Zhao et al., 2010; Zhu et al., 2010b). Thus, the expression of GadA/B represents the most effective pathway for acid tolerance in this bacterium. Our experimental results showed that the acid tolerance of wild-type strain 301 was significantly higher than that of strains 301/pSf-R27 and 301/pAK-sfh, indicating that the presence of plasmid pSf-R27 and the expression of Sfh reduced the expression of GadA/B (Figure 5D). Interestingly, strain 2457T is an efficient diarrheal pathogen, surviving in the acidic gut environment along with other *S. flexneri* strains despite having almost undetectable levels of GadA/B. We suspect that this strain has elevated expression of AR1 or other acid resistance pathways, which have developed to compensate for the low expression of GadA/B. Moreover, the levels of GadA/B in strain 301 could not be reduced to the levels observed in strain 2457T after transfection of pSfR27 or pAK-sfh. However, a decreased survival rate was detected for strains 301/pSf-R27 and 301/pAK-sfh, which suggests that strains 301 and 2457T have different dependencies on GadA/B for acid resistance. We therefore speculate that GadA/B may play a more important role in the acid resistance of strain 301 than in strain 2457T. This study furthers our understanding of the acid tolerance of *S. flexneri*, and through the discovery of the novel regulatory protein Sfh, provides a new way to study the acid resistance mechanisms of enterobacteria.

## Author contributions

HW and LZ conceived, organized, and interpreted experiments. CN generated the bulk of the results and wrote the manuscript. HL, XRL, XML, and RW. performed the experiments. DW and CP assisted in performing and interpreting experiments. XKL and EF analyzed the results and datasets. WX and XQL provided advice and expertise.

### Conflict of interest statement

The authors declare that the research was conducted in the absence of any commercial or financial relationships that could be construed as a potential conflict of interest.
