# Supplementary material for: An H-NS Family Protein, Sfh, Regulates Acid Resistance by Inhibition of Glutamate Decarboxylase Expression in Shigella flexneri 2457T
Source: Front Microbiol. 2017 Oct 5;8:1923. doi: 10.3389/fmicb.2017.01923 (PMC5633597; doi:10.3389/fmicb.2017.01923)

**Supplemental data**

FIG. S1. **Construction of the 301/R27L transconjugant**.

Cm^r^, chloramphenicol resistance; Nal^r^, nalidixic acid resistance; Sm^r^, streptomycin resistance

FIG. S2. **Results of PCR screening of R27 in 301 and 2457T strains.**

Genes ipaD and ipaB are located on the large virulent plasmid ( pCP301 and pINV-2457T). R27_65 and R27_139 primer sets are located on pSf-R27 plasmid. Lane M, DNA marker (CWBio, China).

Figure S1


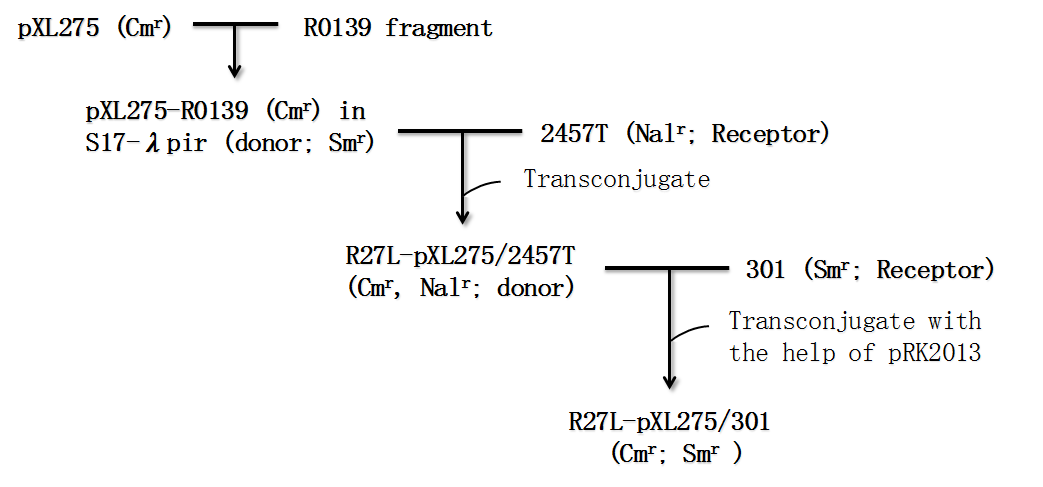


Figure S2


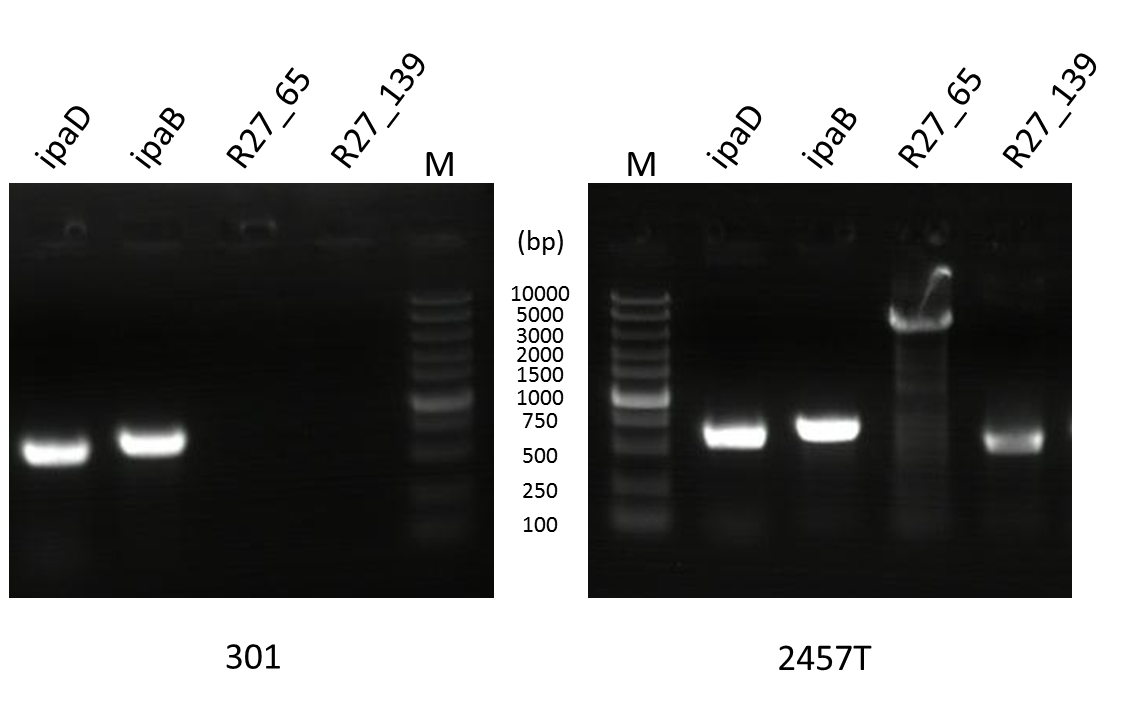


| Primer name | Primer sequence (5’-3’) |
| --- | --- |
| ipaD-F | GCCGATTGTTCCACCTAAT |
| ipaD-R | AGTTCTCATCCTGTAAGTTCC |
| ipaB-F | TGAAGGACTAACCAGAGACTAT |
| ipaB-R | ATAGCATCCGTAACCATAAGAG |
| R27_65-F | TTTATGATGACGGCGGAAGT |
| R27_65-R | AGTTGGGTTGCTGTAGGGAT |
| R27_139-F | TGAAAGATACTGGGAAATAG |
| R27_139-R | TTTCAACGCTTACCCATTCC |

TABLE S1. *Primers used in this study*


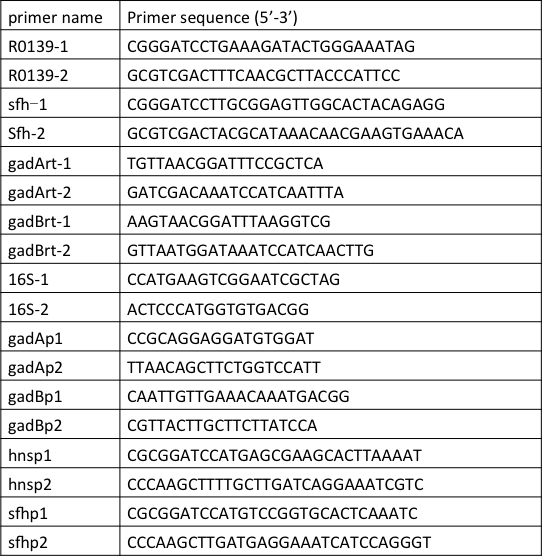

Supplement: Supplementary file 1 [file DataSheet1.DOC]
